# Resistance of endothelial cells to SARS-CoV-2 infection *in vitro*

**DOI:** 10.1128/jvi.01205-25

**Published:** 2025-12-05

**Authors:** Blerina Ahmetaj-Shala, Thomas P. Peacock, Laury Baillon, Olivia C. Swann, Hime Gashaw, Arjun Rustagi, Wendy S. Barclay, Jane A. Mitchell

**Affiliations:** 1 National Heart and Lung Institute, Imperial College London 4615, London, United Kingdom; 2 Department of Infectious Disease, Imperial College London 4615, London, United Kingdom; 3 Department of Medicine, University of California 8785, San Francisco, California, USA; Loyola University Chicago - Health Sciences Campus, Maywood, Illinois, USA

**Keywords:** endothelial cells, COVID-19, SARS-CoV-2, infection, B.1.1.7

## Abstract

The secondary thrombotic/vascular clinical syndrome of COVID-19 suggests that SARS-CoV-2 infects the endothelium; however, robust *in vitro* infection of endothelial cells by various strains of SARS-CoV-2 remains to be demonstrated and continues to be debated. Here, we revisit the question of endothelial cell permissiveness to SARS-CoV-2 using isolated endothelial cells (from the lung, aorta, and endothelial cell progenitors), and additionally, to overcome limitations associated with cultured cells, using native endothelial cells within living precision cut human lung slices and single-cell RNA sequencing to track viral presence. Cellular infection in endothelial monocultures was determined using fluorescence imaging. Mediator release was measured by ELISA, and gene expression was assessed by RT-qPCR. Infection in lung slices was determined using single-cell RNA sequencing, capturing molecular identifiers that aligned to the SARS-CoV-2 viral genome (for lung slices). Each cultured endothelial cell type displayed functional viral responses by increased release of IP-10 when stimulated with Poly-IC (TLR3) or Imiquimod (TLR7/8). Compared to nasal epithelial cells, endothelial cells expressed low or undetectable levels of *ACE2* and showed susceptibility to Ebola and Vesicular Stomatitis Virus glycoprotein-expressing pseudoviruses but not live SARS-CoV-2. Importantly, native endothelial cells within human lung slices displayed minimal infectability with SARS-CoV-2. To our knowledge, this is the first study to demonstrate that neither cultured nor native human endothelial cells are particularly, directly permissive to SARS-CoV-2, likely due to the lack of sufficient *AEC2* expression. These observations confirm that the vascular inflammation and cardiovascular consequences of COVID-19 are largely an indirect result of paracrine inflammatory responses.

**IMPORTANCE:**

SARS-CoV-2 is recognized not only for its acute effects and links with cardiovascular events but also for its ability to cause long COVID syndrome, which is now a major concern particularly since its long-term implications remain poorly understood. Revisiting endothelial cell permissivity to SARS-CoV-2 is therefore critical in this setting. We show that SARS-CoV-2, and several strains, do not infect cultured different types of endothelial cells cultured alone or native endothelial cells in situ in human lung tissue. Our findings are in line with the idea that vascular inflammation and thrombosis seen in COVID-19 are independent of direct endothelial cell infection and likely to be mediated by factors released by adjacent infected cells or circulating systemic inflammatory mediators. Our work also suggests that where viremia occurs, SARS-CoV-2 passes through the endothelium, facilitated by loss of barrier function because of local inflammation at the site of infection.

## INTRODUCTION

COVID-19 was one of the most important clinical challenges the scientific community had faced in recent memory. The development of successful vaccines dramatically mitigated the impact of the disease, although the disease remains a serious concern not only as a serious respiratory virus in the acute setting but also in the post-acute phase where serious and chronic conditions have emerged (1). The virus that causes COVID-19, SARS-CoV-2, was first identified in Wuhan, China, in December 2019 (represented by the strain Wuhan-hu-1) (2). Early in 2020, a new variant, D614G, emerged with increased infectivity of SARS-CoV-2, which rapidly became the globally dominant form (3). Subsequently, strains with increased transmissibility, such as B.1.1.7, first identified in the United Kingdom in September 2020, have emerged (4–6). Despite the emergence of various Spike protein mutations, the process of SARS-CoV-2 entry into cells appears to remain unchanged. SARS-CoV-2 enters host airway cells via ACE2 (7), facilitated by the cell-surface protease TMPRSS2 (7) or lysosomal cysteine proteases cathepsin B/L (CTSB and CTSL) (7). It has also been suggested that, as an alternative pathway, SARS-CoV-2 binds to cells via BSG (Basigin; also known as CD147 or EMMPRIN) (8, 9), although firm evidence for BSG as a standalone receptor for SARS-CoV-2 remains the subject of investigation with a recent study noting no “direct” binding of SARS-CoV-2 spike protein to BSG (10).

Initial infection with SARS-CoV-2 occurs via the respiratory epithelium (11). In most people, symptoms are mild, but in a significant minority, COVID-19 progresses to severe disease, and in those that “recover,” symptoms can persist, leading to a syndrome recently defined as “long COVID,” also known as “post-COVID” (12). In severe COVID-19 and in long COVID, multiple organs, including the cardiovascular system, are affected (13–15). This secondary thrombotic/vascular clinical syndrome of COVID-19 suggests that SARS-CoV-2 infects not only the respiratory epithelium but also the endothelium, disrupting barrier function and allowing dissemination to other organs of the body (16, 17). This notion is supported by early reports showing that SARS-CoV-2 infects endothelial cells *in vitro* (18) and *in vivo* (19). However, the *in vitro* studies performed to date all have used cultured cells with both positive (18) and negative (20–22) infection results have been reported. Cultured endothelial cells have limitations since receptors and other key phenotypic pathways can be lost with passage. However, endothelial plasticity is important in viral pathogenesis as SARS-CoV-2 has been shown to induceendothelial-to-mesenchymal transition both *in vitro* (23) and *in vivo* (24). Here, we have revisited the question of primary endothelial cell permissivity to infection by live SARS-CoV-2 using cultured as well as native endothelial cell models.

## RESULTS

**Fig 1:**
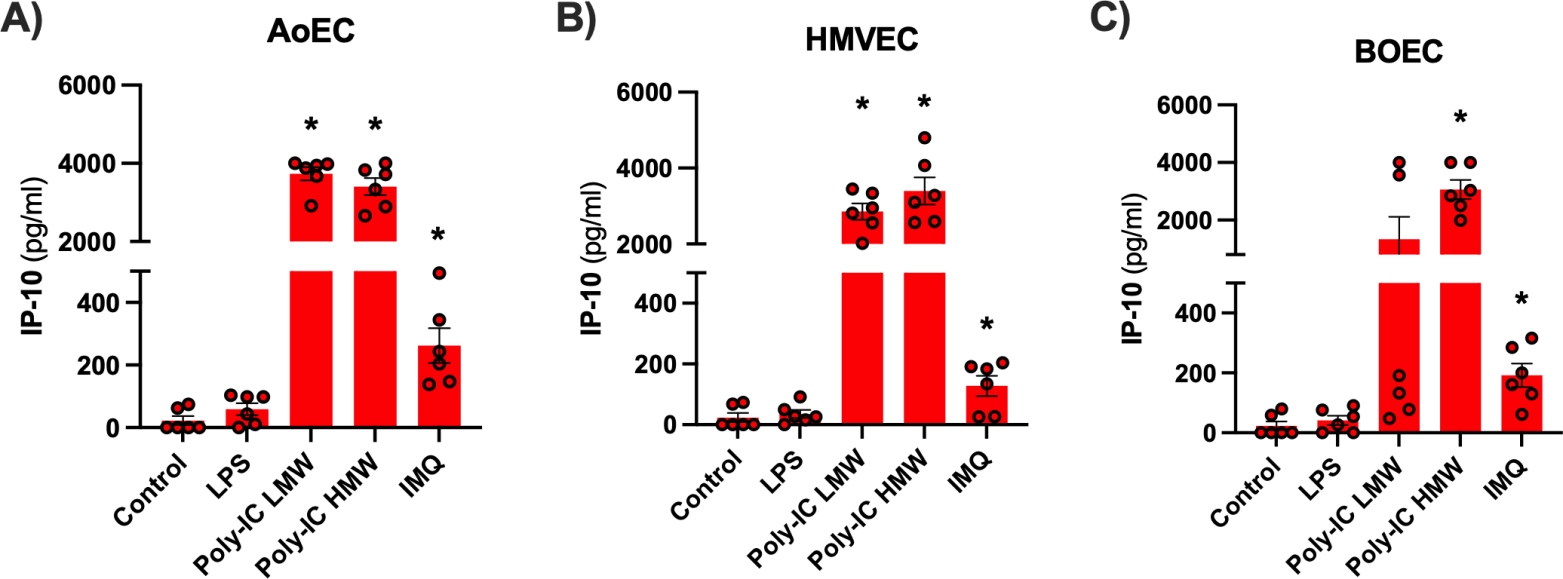
IP-10 expression in human endothelial cells (aortic, microvascular, and blood outgrowth) treated with a panel of PAMPS. IP-10 levels released from human aortic (AoEC; **A**), lung microvascular (HMVEC; **B**), and blood outgrowth (BOEC; **C**) endothelial cells treated for 24 h with control (media only), LPS (1 µg/mL), Poly-IC low or high molecular weight (LMW or HMW, respectively; 10 µg/mL), and Imiquimod (1 µg/mL). Data are shown as the mean ± SEM from *n* = 6 wells from *n* = 3 donors separate donors for AoEC, HMVEC, and BOEC. Data were analyzed using a paired one-way ANOVA and Dunnett’s post hoc test compared to control, with significance denoted as *P < 0.05.

Innate immune responses stimulated by viral entry are mediated by pattern recognition receptors including TLR3 and TLR7/8 (25, 26). We found that each of the endothelial cell types tested has the sensing pathways needed to respond to viral pathogens as indicated by increased release of IP-10 when cells were stimulated with Poly-IC (TLR3) or Imiquimod (TLR7/8) (Fig. 1). Similar results were obtained for IL-6 and IL-8 cytokines (Fig. S1) or when cells were primed with IL-1β (Table S1). These results confirm that if SARS-CoV-2 enters or infects endothelial cells, they could mount an authentic immune/inflammatory response.

**Fig 2:**
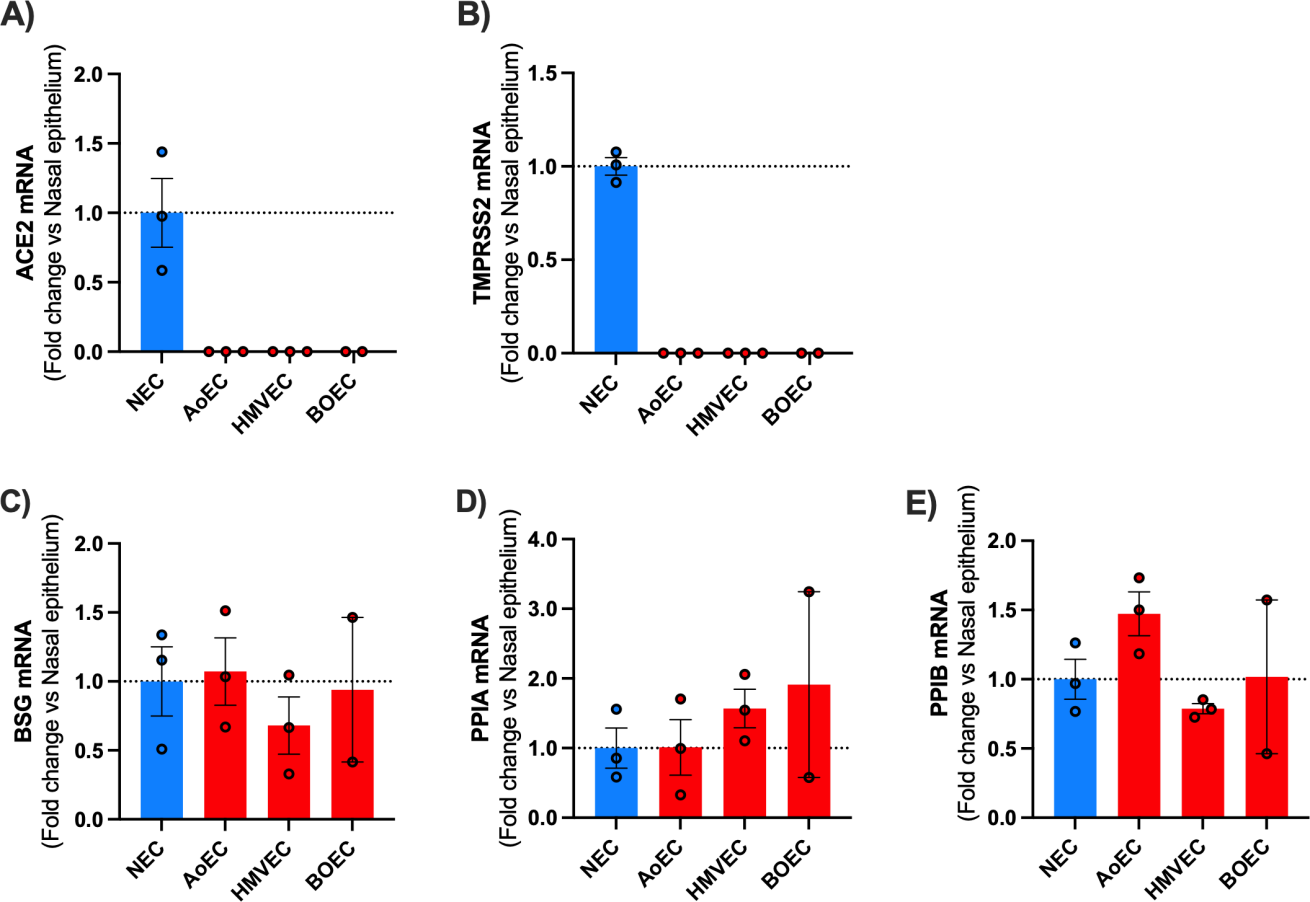
mRNA expression of ACE2, *TMPRSS2*, *BSG*, *PPIA*, and PPIB in human nasal epithelial cells (NECs) and endothelial cells (aortic, microvascular, and blood outgrowth). Expression levels for the genes *ACE2* (**A**), *TMPRSS2* (**B**), *BSG* (**C**), *PPIA* (**D**), and *PPIB* (**E**) were obtained from aortic (AoEC), microvascular (HMVEC), and blood outgrowth (BOEC) endothelial cells and NECs. Data for each donor were normalized using the average of the housekeepers (18S and Gapdh) and analyzed using a comparative Ct method (2ΔΔCt). Data are shown as the mean ± SEM fold change compared to nasal epithelium (*n* = 3 wells using cells from two donors) for AoEC (*n* = 3 wells using cells of three separate donors), HMVEC (*n* = 3 wells using cells of three separate donors and BOECs (*n* = 2 wells using cells of two separate donors).

Next, we investigated the levels of expression of SARS-CoV-2 entry genes in each endothelial cell type. We found that endothelial cells expressed low or undetectable levels of *ACE2* and *TMPRSS2* compared to nasal epithelial cells*,* in line with our previous work (27) (Fig. 2; Table S2). This suggests that if endothelial cells are susceptible to infection with SARS-CoV-2, it may be via an ACE2-independent pathway. BSG has been proposed as a putative SARS-CoV-2 receptor. Endothelial cells and nasal epithelial cells expressed comparable levels of *BSG* and Cyclophilin A*/B (PPIA/B*, *PPIA*, and *PPIB*), which are potential co-ligands for pathogen entry to cells via the BSG pathway (28–31) (Fig. 2).

**Fig 3:**
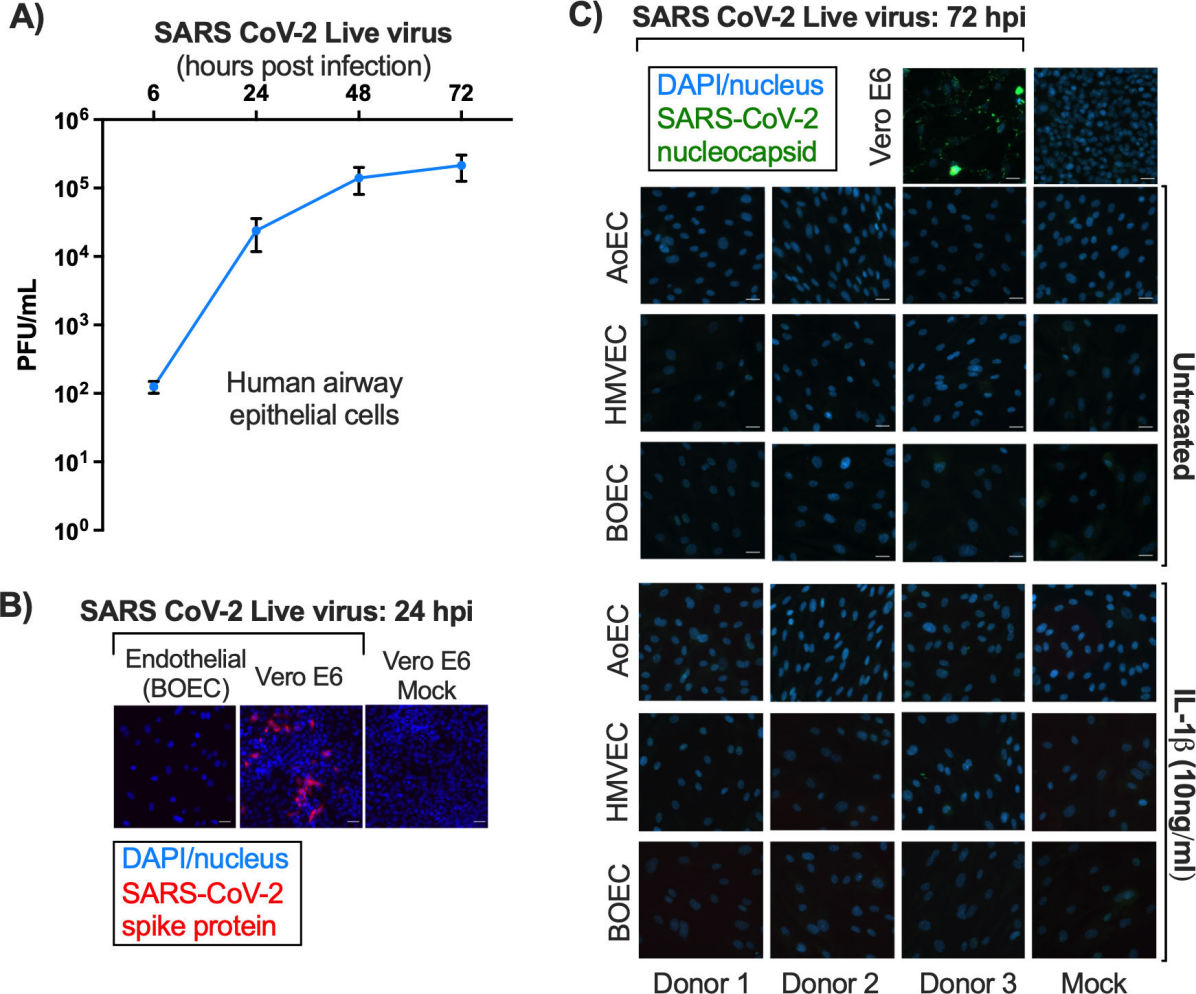
SARS-CoV-2 virus infection in human airway epithelial cells in air:liquid interface, Vero E6, and endothelial cells. Human airway epithelial cells grown in an air:liquid interface (MucilAir) were infected with SARS-CoV-2 live virus (MOI = 0.1). Infectious virus released to the apical side of the epithelium was determined over time (6, 24, 48, and 72 h post-infection) (**A**). In separate studies, the levels of SARS-CoV-2 nucleocapsid or spike protein in Vero E6 and endothelial cells (treated with media only [untreated] or IL-1β [10 ng/mL; 3 h]) at 24 (**B**) and 72 (**C**) h post-infection with SARS-CoV-2 (MOI = 0.1) were determined using fluorescent imaging. Mock controls (media only) experiments were run simultaneously using each endothelial cell line. Data are shown as *n* = 3 (pooled donors) for Mucilair cells (**A**) and *n* = 3 (separate donors) for human aortic (AoEC), lung microvascular (HMVEC), and blood outgrowth endothelial cells (BOECs). Data are shown as mean ± SEM for (A) and representative images shown for (B) and (C) (scale bar = 25 µm).

As we have described previously (32), nasal epithelial cells grown in liquid:air interface culture were susceptible to infection with SARS-CoV-2 (Fig. 3A). Using similar conditions, we inoculated endothelial cells and permissive Vero E6 cells with the same multiplicity of SARS-CoV-2 (Fig. 3B). By indirect immunofluorescence microscopy, viral antigens (nucleocapsid protein or spike) were observed in Vero E6 cells (indicating virus replication) but not in the endothelial cell types at 24 or 72 h post-inoculation (Fig. 3B and C; Fig. S2). These results suggest that endothelial cells are not permissive for SARS-CoV-2 infection. From these data, however, it is unclear if this observed block exists at the level of virus entry or at a post-entry step preventing replication and expression of virus genes.

**Fig 4:**
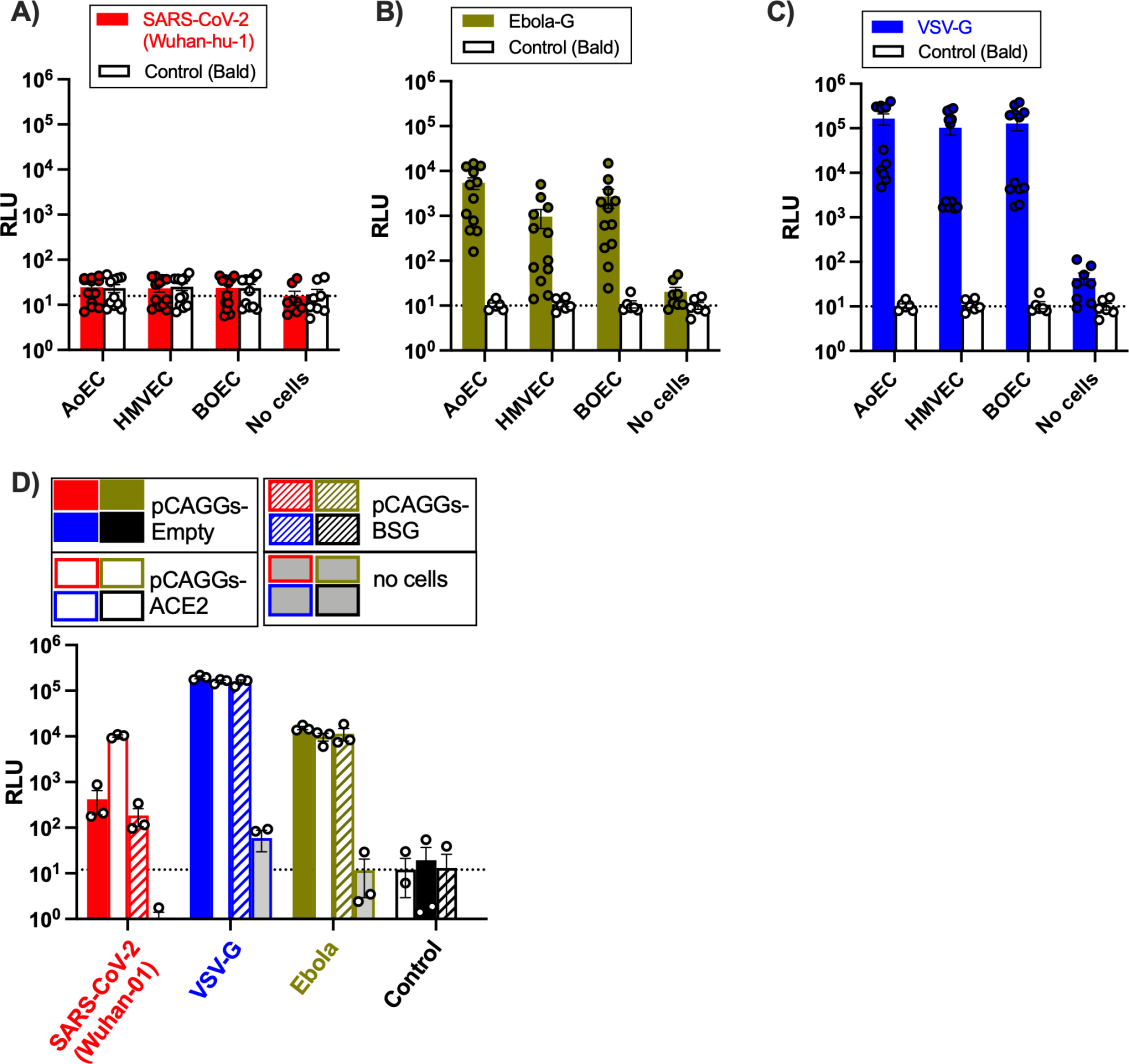
Pseudovirus infection with SARS-CoV-2 (**A**), Ebola (**B**), vesicular stomatitis virus G (VSV-G) (**C**) in endothelial cells or SARS-CoV-2 in kidney embryonic kidney (HEK 293T) cells transfected with ACE2 or BSG (**D**). Cell entry by SARS-CoV-2 (Wuhan-hu-1) (**A**), Ebola (**B**), and VSV-G (**C**) pseudovirus levels was quantified by Luciferase Assay 48 h post-transduction. Overexpression of ACE2-FLAG and CD147/BSG in HEK 293T cells was confirmed by western blot (**D**). Data were from *n* = 12 wells using cells from three separate donors in experiments performed on two separate occasions for aortic (AoEC), microvascular (HMVEC), and blood outgrowth (BOEC) (**A–C**); ACE2-HEK-2932T and BSG-HEK-2932T are *n* = 3. Data are expressed as individual values and as mean – SEM. Dotted lines represent background signal (mean control/bald [no cells or empty]).

To investigate this further, we performed studies using lentiviral pseudoviruses displaying SARS-CoV-2 spike protein (Wuhan-hu-1) or glycoproteins of vesicular stomatitis virus (VSV-G), which shows broad cell-type tropism, or Ebola virus, which has previously been shown to efficiently infect endothelial cells (33) (Fig. 4). Endothelial cells were not susceptible to SARS-CoV-2 spike-mediated entry, but pseudoviruses expressing Ebola-G or VSV-G were able to enter each type of endothelial cell used (Fig. 4A through C). These observations are in line with low-level ACE2 expression in endothelial cells and suggest that the high levels of BSG expression do not compensate to allow infection with SARS-CoV-2. To address this assumption, we investigated SARS-CoV-2 spike protein pseudovirus entry into HEK 293T cells transiently transfected with either ACE2 or BSG (Fig. 4D; Fig. 2). HEK 293T cells expressing ACE2 showed high susceptibility to all three pseudoviruses. By contrast, HEK 293T cells expressing BSG were susceptible to Ebola-G and VSV-G but not SARS-CoV-2 spike-mediated pseudovirus entry.

**Fig 5:**
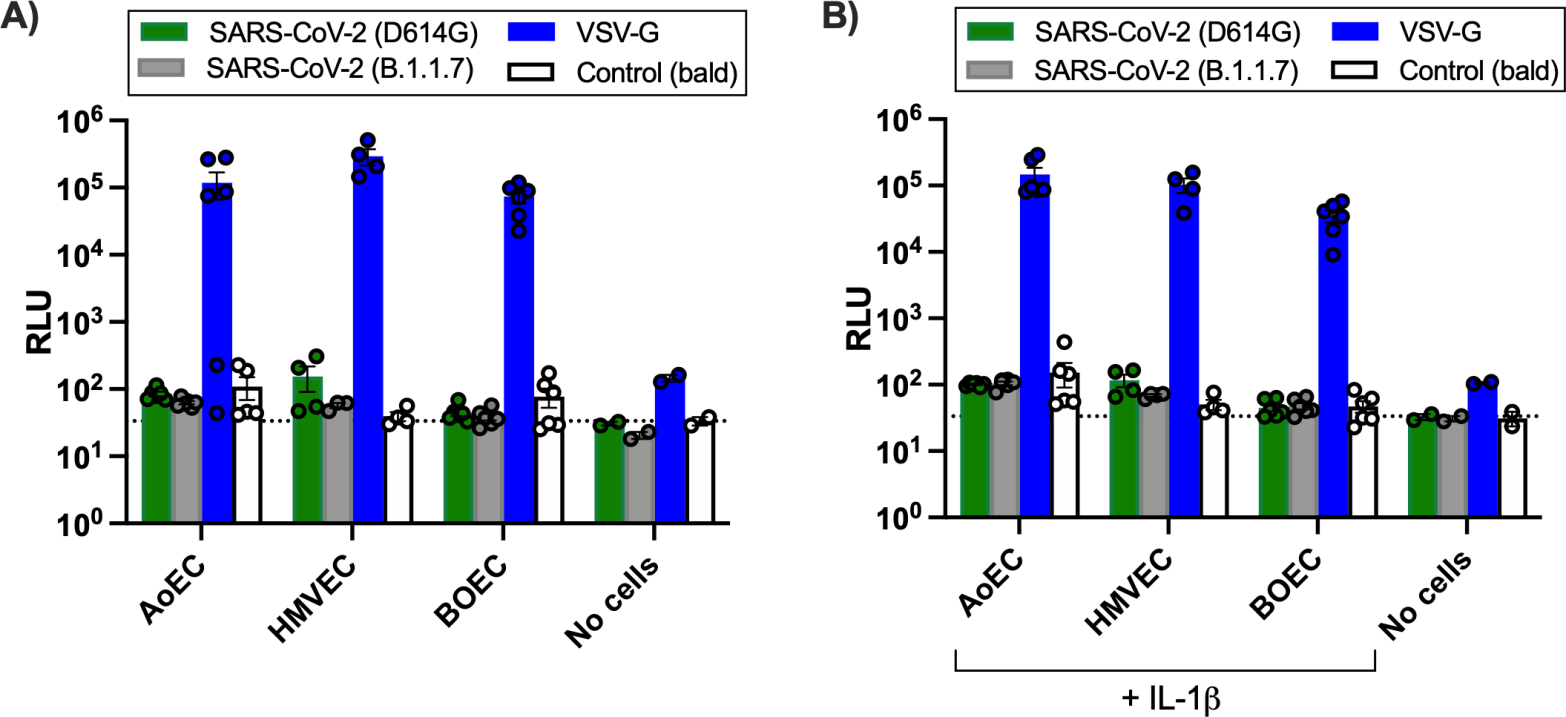
Susceptibility of endothelial cells treated with control (**A**) or IL-1β (**B**) to pseudoviruses expressing SARS-CoV-2 spike (D614G or B.1.1.7), vesicular stomatitis virus glycoprotein (VSV-G) or control (bald). Cell entry by SARS-CoV-2 spike (D614G or B.1.1.7), VSV-G, or control (bald) pseudovirus entry was quantified by Luciferase Assay 48 post-infection in endothelial cells untreated (**A**) or treated with IL-1β (10 ng/mL) for 3 h prior to infection (**B**). Data were from *n* = 4–6 wells from *n* = 3 donors of aortic (AoEC) and blood outgrowth (BOEC) and *n* = 2 donors microvascular (HMVEC). Data are expressed as individual values and mean ± SEM. Dotted lines represent background signal (mean control/bald [no cells or empty]).

To investigate whether inflammation might facilitate viral entry of SARS-CoV-2 into endothelial cells, cells were primed with IL-1β before inoculation with virus. At 24 h post-inoculation, the IL-1β primed endothelial cells released IL-6 and IL-8, but not IP-10, demonstrating a positive inflammatory response to IL-1β had been induced (Fig. S3). However, under these conditions, each of the endothelial cell types remained refractory to SARS-CoV-2 infection as indicated by the lack of virus antigen expression (Fig. S4). Similar results were found when cells were stimulated with IL-6 (data not shown). To investigate whether the other SARS-CoV-2 strains might have endothelial tropism, we compared entry of pseudoviruses expressing the variant SARS-CoV-2 B.1.1.7 or D614G spike glycoproteins. In the presence or absence of IL-1ß, endothelial cells were not susceptible to entry by any of the SARS-CoV-2 strains, but as before, pseudoviruses expressing VSV-G were able to efficiently enter endothelial cells (Fig. 5; Fig. S5).

**Fig 6:**
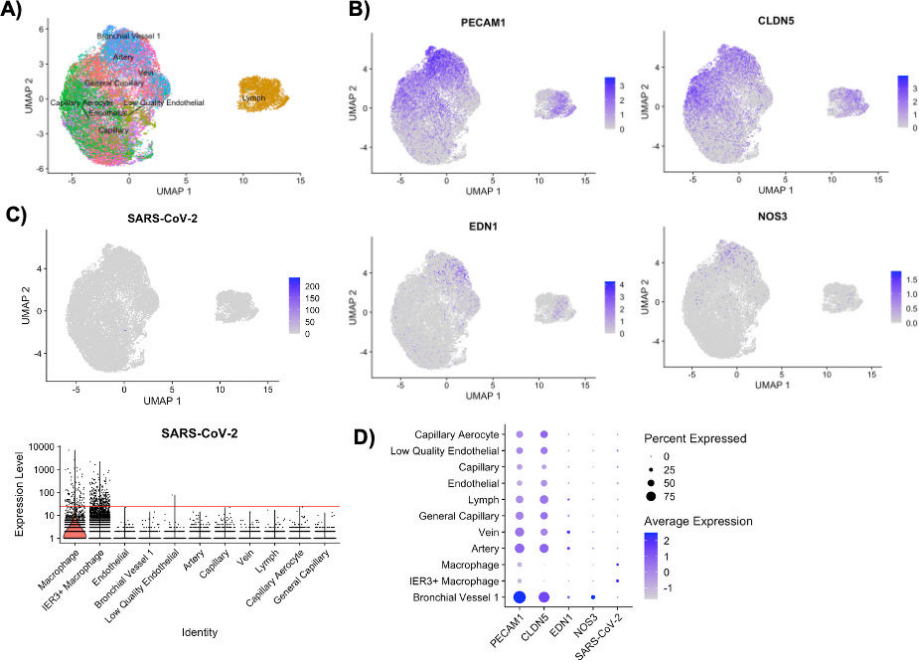
Endothelial cells in human lung slice cultures were not found to take up SARS-CoV-2. From the total data set, only endothelial cells were selected, projected onto UMAP space, and plotted, with labels from the Human Lung Cell Atlas (**A**). Using normalized expression levels and the same projection, the same cells were plotted for *PECAM1*, *CLDN5*, *EDN1*, and *NOS3* (**B**), illustrating expression of canonical endothelial lineage markers. The same cells and projection were then plotted for absolute expression of SARS-CoV-2 (**C**, top), while a violin plot (**C**, bottom) shows the absolute expression of SARS-CoV-2, on a logarithmic scale, for each endothelial cell subtype, with macrophages and IER3+ macrophages added for comparison. The red line marks 25 counts of SARS-CoV-2 RNA. A dot plot summarizing relative average expression of each marker for each cell type is shown in (**D**).

Finally, we turned to published data from cultured human lung slices, which contain primary endothelial cells in the context of the lung environment, that were infected with SARS-CoV-2 *in vitro* and subjected to single-cell RNA sequencing (34, 35). In both studies, endothelial cells rarely contained SARS-CoV-2 transcripts and were not considered likely target cells. To confirm, we pulled and analyzed the publicly available data from Wu et al. (34) and found negligible infection of endothelial subtypes (Fig. 6).

## DISCUSSION

There is mounting speculation that the vascular and thrombotic sequela associated with severe COVID-19 are a result of endothelial cell infection with SARS-CoV-2 (36). We have directly addressed the question of “are endothelial cells permissive to infection with SARS-CoV-2 virus?” To address this question, we have analyzed both cultured endothelial cell monolayers and endothelial subtypes in their native setting within intact human lung slices.

In our reanalysis of lung slice scRNA-seq data from Wu et al. (34), which agrees with another lung slice study (35), primary pulmonary vascular endothelial cells were rarely found to have SARS-CoV-2 transcripts. This is in stark contrast to the robust detection of SARS-CoV-2 transcripts in lung macrophages in both studies. A limitation of scRNA-seq on the lung slice system is that live, singlets from cell types that form tight junctions (e.g., vascular endothelium and airway epithelium) are challenging to recover, especially after the stresses of explantation, slicing, culture, infection, enzymatic digestion, and mesh filtration. That said, the endothelial compartment in Fig. 6 includes 21,639 cells across four donors (with additional macrophages included in Fig. 6C, bottom, and Fig. 6D). In comparison, 25,095 epithelial cells were recovered in the same data set, and of those, several SARS-CoV-2-infected cells were identified (34), which argues against undersampling.

Our results were unambiguous and interpreted alongside data from robust positive control cells infected with live virus and pseudoviruses from different SARS-CoV-2 Spike mutations. Taken together, our data suggest that endothelial cells are not susceptible to infection with SARS-CoV-2, most likely because they express insufficient levels of ACE2, which is also in agreement with other studies (21, 22, 27). It should be noted, however, that several studies using immunohistochemistry show that some subsets of vascular cells, including endothelial cells, express ACE-2 (37, 38).

Our findings that endothelial cells remain non-susceptible to infection with SARS-CoV-2 despite expressing high levels of BSG suggest that the BSG pathway is not a functional entry pathway for SARS-CoV-2 in endothelial cells. This idea is confirmed from our studies using HEK 293T cells transiently transfected with BSG which, in contrast to ACE2, did not make cells susceptible to SARS-CoV-2 spike-mediated entry. Importantly, we show that even in the UK SARS-CoV-2 spike variant, B.1.1.7, which shows higher mortality rates than previous strains (5), endothelial cells are not susceptible to entry, excluding the hypothesis that this pathogenicity is caused by increased endothelial cell tropism. Further studies such as plaque assays or subgenomic RNA evaluation are important to analyze the presence or absence of viral replication. Furthermore, our study emphasizes replication as a key indicator of infection; however, there is a need for a more nuanced interpretation of infection markers across different investigative settings.

In conclusion, and in agreement with Wang et al. (20) and Hui et al. (39), our results suggest that the vascular dysfunction and thrombosis seen in severe COVID-19 is a result of factors released by adjacent infected cells (e.g., epithelial cells or immune cells such as macrophages) and/or circulating, systemic inflammatory mediators or spike protein/structural components rather than of direct infection by SARS-CoV-2. Where viremia occurs in COVID-19, SARS-CoV-2 may pass through the endothelium facilitated by loss of barrier function, as a result of local inflammation at the site of infection.

## MATERIALS AND METHODS

### Biosafety statement

All work performed was approved by the local genetic manipulation (GM) safety committee of Imperial College London, St. Mary’s Campus (center number GM77), and the Health and Safety Executive of the United Kingdom, under reference CBA1.77.20.1.

### Cells

Primary human endothelial cell lines including blood outgrowth (obtained in house [40]), aortic and lung microvascular cells (obtained from Lonza, UK) were maintained in uncoated plates in Endothelial Cell Growth Medium-2 (EGM-2; Promocell, Germany or Lonza) or Microvascular EGM-2 (Lonza) respectively. Nasal epithelial cells (Promocell) were maintained in Airway Epithelial Growth Media (Promocell) and differentiated nasal epithelial cells (MucilAir) (Epithelix, Switzerland) were grown in air-liquid interface culture using MucilAir Culture Medium (Epithelix). All endothelial/epithelial cell media were supplemented with their appropriate BulletKits and 1% penicillin-streptomycin (P/S) (Gibco, UK). African green monkey (Vero E6) cells and human embryonic kidney cells (HEK 293T) (both from ATCC) were cultured in Dulbecco’s modified Eagle’s medium (DMEM), supplemented with 10% fetal bovine serum (FBS), 1% non-essential amino acids (NEAA), and 1% P/S (Gibco). Transient transfections of different receptors in HEK 293T were performed as described elsewhere and maintained in HEK 293T media supplemented with 1 µg/mL of puromycin (41). Treatment protocols (except for those using MucilAir cells) were conducted using “treatment media” (EGM-2 medium (Promocell) supplemented with 2% FBS (Promocell) and 1% P/S. For all experiments, cells were used between passages 4 and 6 and maintained at 37°C, 5% CO_2_.

### RT-qPCR

Endothelial and nasal epithelial cells were plated in duplicate wells on uncoated six-well plates in their cell-specific media (see above) and grown to confluence. The day before RNA extraction, media were replaced with the treatment media. After 24 h, cells were washed with phosphate-buffered saline (PBS), duplicate wells combined, and RNA extracted using RNeasy Extraction kit (Qiagen, UK). RNA was converted to cDNA using the iScript cDNA synthesis kit (Bio-Rad, CA, USA). Gene expression levels were determined using a TaqMan expression assay, with the following primers (ThermoScientific, UK); ACE2 (Hs01085333_m1), TMPRSS2 (Hs00237175_m1), BSG (Hs00936295_m1), PPIA (Hs04194521_s1), and PPIB (Hs00168719_m1). Genes were quantified relative to housekeeping genes (GAPDH and 18S) by the comparative Ct method.

### SARS-CoV-2 live virus infection studies

Infection studies with MucilAir airway epithelial cells were performed as previously described (41). Briefly, SARS-CoV-2/England/IC19/2020 (IC19) (42) was used to infect at a multiplicity of infection (MOI) of 0.1. At each time point in the infection, virus was collected from the apical surface of the cultures in PBS and quantified by plaque assay on Vero E6 cells as described elsewhere (41).

For endothelial cell infection, cells were plated on sterile round 16 mm diameter coverslips in 12-well plates (5 × 10^4^ cells/well) without coating in their usual media (see above) and allowed to settle overnight. Control Vero E6 cells were plated to achieve approximately 80% confluency. The following day, media were removed, and cells were washed in PBS. For endothelial cells, treatment media were added either alone (untreated/control) or with IL-1β (10 ng/mL) for 3 h. Meanwhile, IC19 (42) was diluted in serum-free DMEM, 1% NEAA, 1% P/S, to an MOI of 0.1. After 3 h treatment with either media alone or IL-1β, media were replaced with IC19 containing inoculum and incubated at 37°C for 1 h. Inoculum was then removed and replaced with treatment media, and cells were maintained until 24, 48, or 72 h post-infection. At the appropriate time point, treatment media were removed, and cells were fixed in 4% paraformaldehyde for 30 min.

### Fluorescent imaging

Infected cells were stained using primary antibodies against spike protein (S) (Mouse monoclonal, Gene tex [1A9]) or nucleoprotein (N) (Rabbit monoclonal, Sino Biological) and secondary antibodies (anti-rabbit 488 and anti-mouse 594) and DAPI. Images were acquired using a Zeiss Axiovert 135 TV microscope (Fig. 3B) or a Zeiss Cell Observer widefield microscope (Fig. 3C). Representative fields were captured and processed in an identical manner across each experiment either as a single plane of focus (Fig. 3B) or as *Z* stacks presented as maximum intensity projections (Fig. 3C). For quantitative analysis, images were blinded and independently scored between 1 and 5, where 1 = 0–2, 2 = 3–5, 3 = 6–8, 4 = 9–10, and 5 ≥ 10 nucleocapsid-stained cells.

### Lentiviral pseudovirus studies

Lentiviral pseudotypes were generated as previously described (41). Target cells plated in duplicate in 96-well plates (1 × 10^4^/ well) were allowed to settle overnight in their appropriate growth media. The next day, media were replaced with treatment media alone (untreated/control) or with IL-1β (10 ng/mL) for 3 h. Media were replaced with pseudovirus-containing supernatants for 2 h before replacing with treatment media for 48 h before adding lysis buffer (Promega). Luminescence was read on a FLUOstar Omega plate reader (BMF Labtech) using the Luciferase Assay System (Promega).

### Western blot

Western blot analysis of HEK 293Ts transfected with different receptors was performed as previously described (41). Briefly, cells were lysed in RIPA buffer and mixed with 4× Laemmli sample buffer (Bio-Rad) with 10% β-mercaptoethanol. Protein samples were run on a 4–15% mini-PROTEAN TGX SDS-PAGE gel (Bio-Rad) and transferred onto a nitrocellulose membrane by semi-dry transfer. Membranes were probed with the primary antibodies: mouse anti-FLAG (Sigma: F1804), mouse anti-tubulin (Abcam; ab7291), and rabbit anti-CD147 (Abcam; ab108308). Near-infra-red (NIR) secondary antibodies, IRDye 680RD Goat anti-mouse (Abcam; ab216776) and IRDye 800CW Goat anti-rabbit (Abcam; ab216773), were subsequently used. Western blots were visualized using an Odyssey Imaging System (LI-COR Biosciences).

### IL-6, IL-8, and IP-10 ELISA

IL-6, IL-8, and IP-10 were measured using duo set ELISAs from R&D Systems according to the manufacturer’s instructions. For IL-6 and IL-8 measurements, samples collected from SARS-CoV-2 live virus infection studies were used. For IP-10 measurements, all three primary endothelial cell lines were plated in 96-well plates, allowed to settle overnight. The next day, cells were left untreated or primed with IL-1β (10 ng/mL) for 3 h before treating for 24 h with a panel of PAMPs including lipopolysaccharide-*E. coli* (LPS; 1 µg/mL), Poly-IC low and high molecular weight (10 µg/mL), and Imiquimod (1 µg/mL).

### Infected lung slice culture data analysis

The methodology used to culture and infect human lung slices is described in detail elsewhere (35). Annotated scRNA-seq data from Wu et al. (34) was downloaded and queried for endothelial cell infection. To do this, counts data were loaded into R and slotted into a Seurat object. SCTransform using the glmGamPoi method was used to normalize and scale the counts data and regress out mitochondrial and ribosomal RNA, followed by PCA and UMAP. The Harmony package was then run to integrate the data. The object was then filtered to only include cell types labeled as the endothelial compartment (with or without macrophages for comparison) as annotated in Wu et al. based on the Human Lung Cell Atlas (43). UMAP was run on this subsetted object using Harmony embeddings. A cutoff of 25 counts/cell was used in Fig. 6C, bottom, based on background SARS-CoV-2 detection in cells in the data set exposed to virus-inactivating conditions.

### Statistical data analysis

All data were analyzed on GraphPad Prism v8 and are shown as individual data points and/or mean ± standard error of the mean (SEM) as described in the figure legends.

## Data Availability

Data will be made available upon request to the authors.

## SUPPLEMENTAL MATERIAL

Figure S1 jvi.01205-25-s0001.pdf IL-6 and IL-8 expression in human endothelial cells (aortic, microvascular, and blood outgrowth) treated with a panel of PAMPs.

Figure S2 jvi.01205-25-s0002.pdf Blind scoring of SARS-CoV-2 live virus infection in Vero E6 and endothelial cells.

Figure S3 jvi.01205-25-s0003.pdf Effect of IL-1b on release of IL-6 and IL-8 from endothelial cells.

Figure S4 jvi.01205-25-s0004.pdf ACE2 and BSG protein expression in HEK 239T cells.

Figure S5 jvi.01205-25-s0005.pdf Susceptibility of endothelial cells treated with IL-1b to pseudoviruses expressing SARS-CoV-2 spike (A), vesicular stomatitis virus glycoprotein (VSV-G) (B), or Ebola glycoprotein (C).

Table S1 jvi.01205-25-s0006.pdf IP-10 release from endothelial cells stimulated with PAMPs.

Table S2 jvi.01205-25-s0007.pdf Average raw Ct values from qRT-PCR.

ASM does not own the copyrights to Supplemental Material that may be linked to, or accessed through, an article. The authors have granted ASM a non-exclusive, world-wide license to publish the Supplemental Material files. Please contact the corresponding author directly for reuse.
