# Supplementary material for: Resistance of endothelial cells to SARS-CoV-2 infection in vitro
Source: J Virol. 2025 Dec 5;99(12):e01205-25. doi: 10.1128/jvi.01205-25 (PMC12724323; doi:10.1128/jvi.01205-25)
Supplement: Figure S1 — IL-6 and IL-8 expression in human endothelial cells (aortic, microvascular, and blood outgrowth) treated with a panel of PAMPs. [file jvi.01205-25-s0001.pdf]

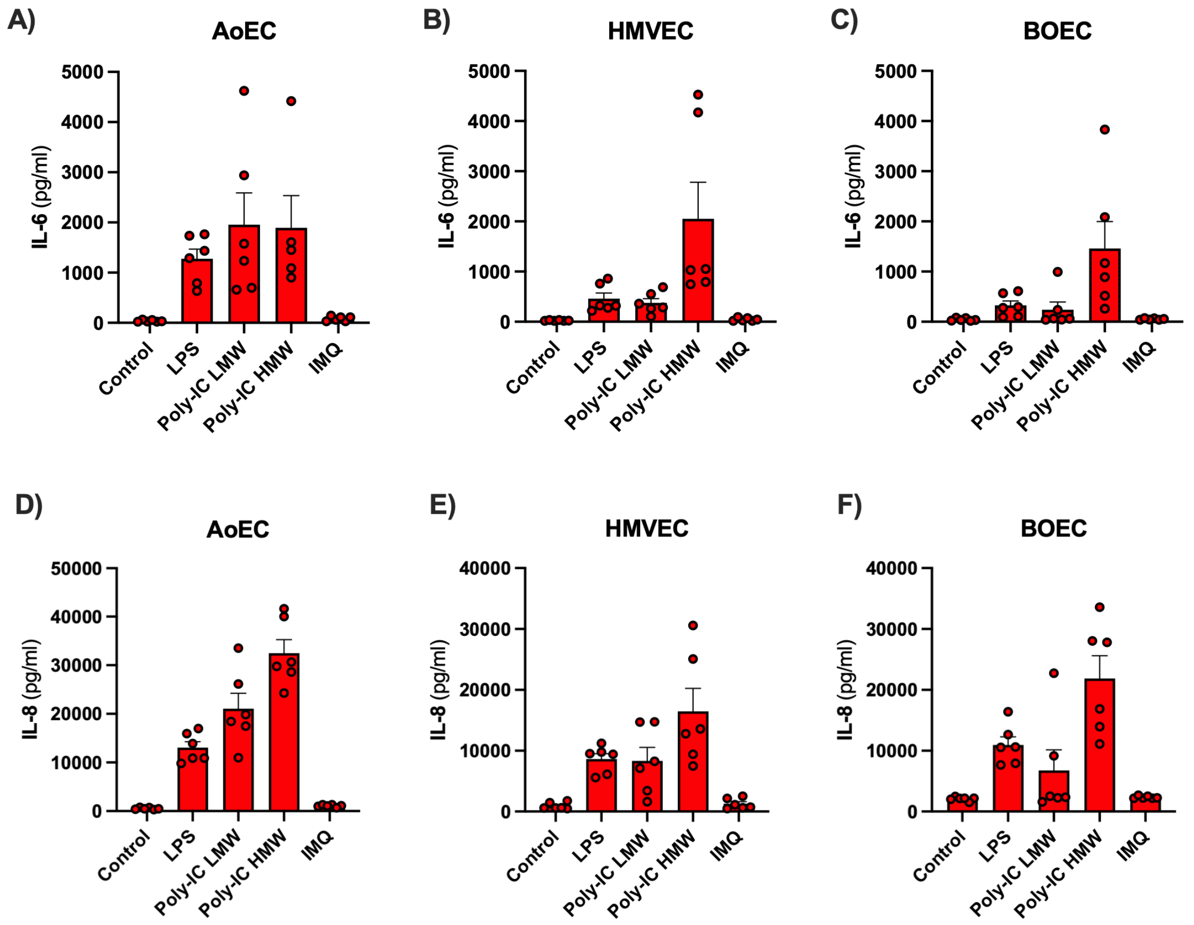

Supplementary Figure 1: IL-6 and IL-8 expression in human endothelial cells (aortic, microvascular and blood outgrowth) treated with a panel of PAMPS. IL-6 (A-C) and IL-8 (D-F) levels released from human aortic (AoEC), lung microvascular (HMVEC) and blood outgrowth (BOEC) endothelial cells treated for 24 hours with control (media only), LPS (1ug/ml), Poly-IC low or high molecular weight (LMW or HMW respectively; 10µg/ml) and Imiquimod (1µg/ml). Data are shown as the mean +/- SEM from n=3 wells from n=3 donors separate donors for AoEC, HMVEC and BOEC.
