# Supplementary material for: Resistance of endothelial cells to SARS-CoV-2 infection in vitro
Source: J Virol. 2025 Dec 5;99(12):e01205-25. doi: 10.1128/jvi.01205-25 (PMC12724323; doi:10.1128/jvi.01205-25)
Supplement: Figure S2 — Blind scoring of SARS-CoV-2 live virus infection in Vero E6 and endothelial cells. [file jvi.01205-25-s0002.pdf]

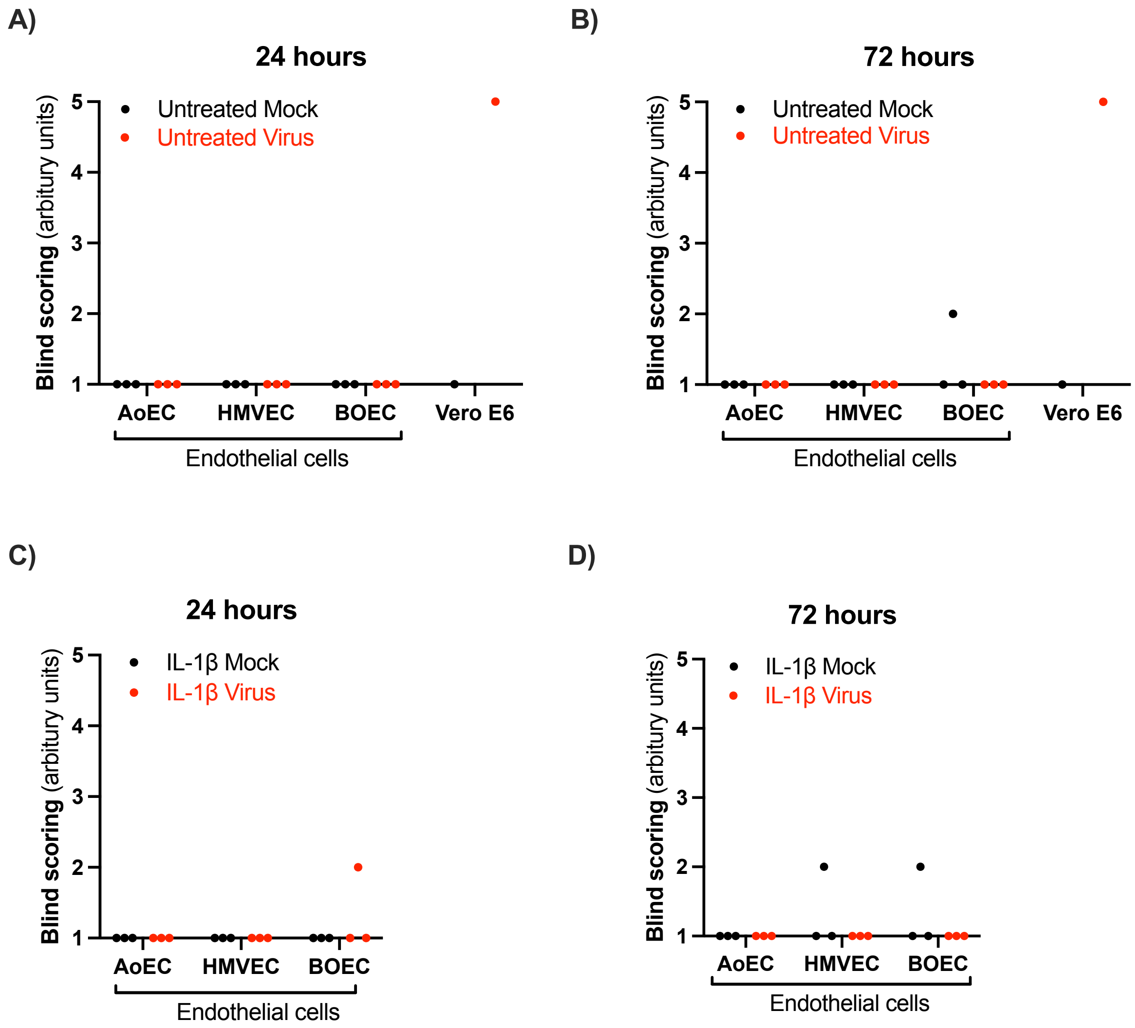

Supplementary Figure 2. Blind scoring of SARS-CoV-2 live virus infection in Vero E6 and endothelial cells. Levels of SARS-CoV-2 of spike protein/ nucleocapsid in Vero E6 and endothelial cells at 24 and 72 hours post infection with SARS-CoV-2 (MOI 0.1) in untreated (A-B) and IL-1ß (10ng/ml; 3 hours) (C-D) were determined using florescent imaging. One representative image was taken and blinded images were scored between 1-5, where 1= 0-2, 2= 3-5, 3= 6-8, 4= 9-10 and 5= >10 virus nucleocapsin/spike protein staining. Data are shown as individual scores for n=3 (separate donors) for human aortic (AoEC), lung microvascular (HMVEC) and blood outgrowth endothelial cells (BOEC) and n=1 for Vero E6 cells (untreated).
