# Supplementary material for: Resistance of endothelial cells to SARS-CoV-2 infection in vitro
Source: J Virol. 2025 Dec 5;99(12):e01205-25. doi: 10.1128/jvi.01205-25 (PMC12724323; doi:10.1128/jvi.01205-25)
Supplement: Figure S3 — Effect of IL-1b on release of IL-6 and IL-8 from endothelial cells. [file jvi.01205-25-s0003.pdf]

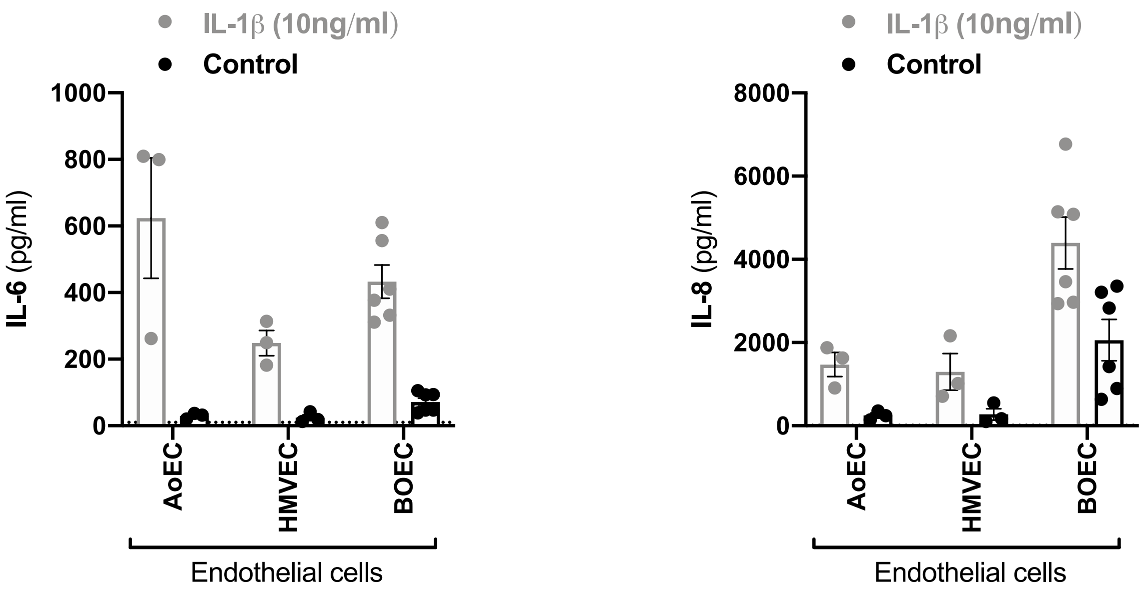


**Supplementary Figure 3: Effect of IL-1β on release of IL-6 and IL-8 from endothelial cells.**

Human endothelial cells (aortic; AoEC, lung microvascular; HMVEC, and blood outgrowth; BOEC) were treated for 3 hours with IL-1β (10ng/ml) before media was replaced with fresh media (EGM-2, 2% FBS) for 24 hours. Media was collected and IL-6 and IL-8 levels measured using ELISA. Data are shown as individual values and mean +/- from n=3 wells using cells from 3 separate donors for AoEC and HMVEC and n=6 wells from 3 separate donors for BOECs.
