# Supplementary material for: Resistance of endothelial cells to SARS-CoV-2 infection in vitro
Source: J Virol. 2025 Dec 5;99(12):e01205-25. doi: 10.1128/jvi.01205-25 (PMC12724323; doi:10.1128/jvi.01205-25)
Supplement: Figure S4 — ACE2 and BSG protein expression in HEK 239T cells. [file jvi.01205-25-s0004.pdf]

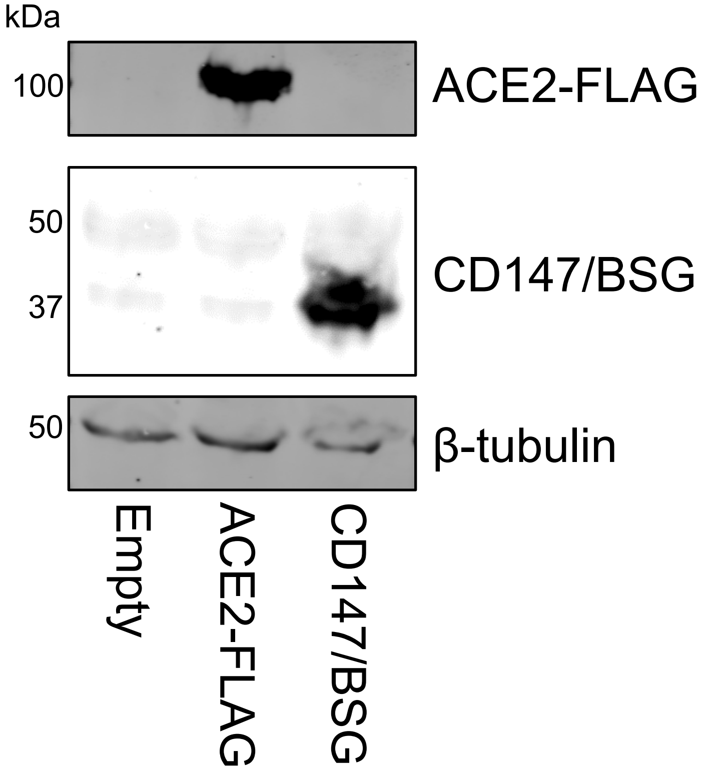

Supplementary Figure 4: ACE2 and BSG protein expression in HEK 239Ts. Overexpression of ACE2-FLAG and CD147/BSG in HEK 293T cells was confirmed by western blot.
