# Supplementary material for: Resistance of endothelial cells to SARS-CoV-2 infection in vitro
Source: J Virol. 2025 Dec 5;99(12):e01205-25. doi: 10.1128/jvi.01205-25 (PMC12724323; doi:10.1128/jvi.01205-25)
Supplement: Figure S5 — Susceptibility of endothelial cells treated with IL-1b to pseudoviruses expressing SARS-CoV-2 spike (A), vesicular stomatitis virus glycoprotein (VSV-G) (B), or Ebola glycoprotein (C). [file jvi.01205-25-s0005.pdf]

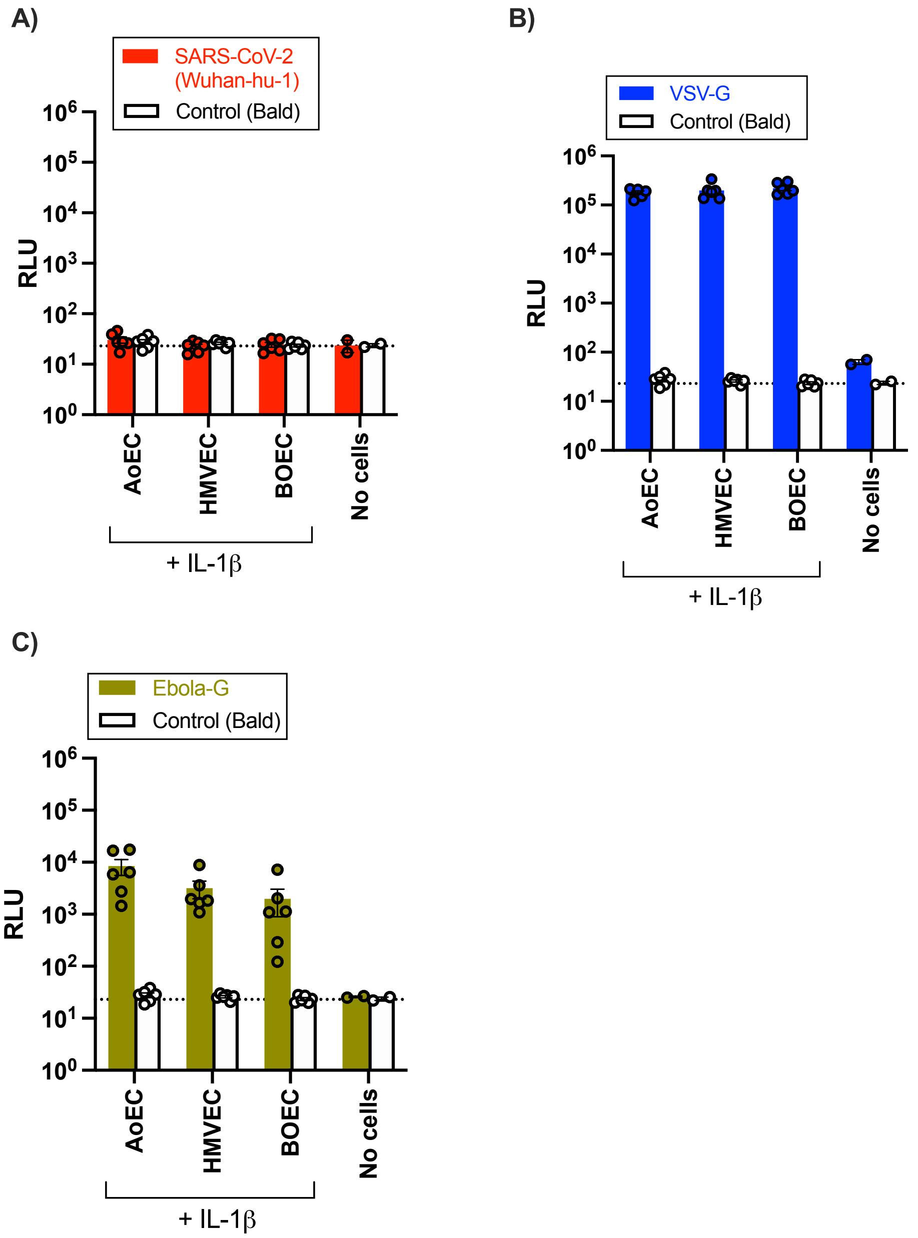

Supplementary Figure 5: Susceptibility of endothelial cells treated with IL-1b **to pseudoviruses expressing SARS-CoV-2 spike (A), vesicular stomatitis virus glycoprotein (VSV-G) (B) or Ebola glycoprotein (C).** Cell entry by SARS-CoV-2 (Wuhan-hu-1) (A) and VSV-G and Ebola (B) pseudovirus entry was quantified by Luciferase Assay 48 hours post infection in endothelial cells treated with IL-1b (10 ng/ml) for 3 hours prior to infection. Data were from n=6 wells from n=3 donors of endothelial cells; aortic (AoEC), microvascular (HMVEC) and blood outgrowth (BOEC). Data are expressed as individual values and mean +/- SEM. Dotted lines represent background signal (mean control/bald (no cells or empty)).
