# Supplementary material for: Resistance of endothelial cells to SARS-CoV-2 infection in vitro
Source: J Virol. 2025 Dec 5;99(12):e01205-25. doi: 10.1128/jvi.01205-25 (PMC12724323; doi:10.1128/jvi.01205-25)
Supplement: Table S1 — IP-10 release from endothelial cells stimulated with PAMPs. [file jvi.01205-25-s0006.pdf]

**Treatment**	**Cell type**	**Untreated (media only)**	**IL-1ß (10ng/ml)**
Control	AoEC	22.8 ± 14.5	51.6 ± 7.9
	HMVEC	23.6 ± 14.9	51.4 ± 6.9
	BOEC	23.2 ± 14.9	49.9 ± 5.8
LPS	AoEC	59.0 ± 19.3	63.2 ± 9.1
	HMVEC	35.4 ± 13.1	61.0 ± 2.8
	BOEC	41.5 ± 15.8	176.6 ± 122.2
Poly-IC LMW	AoEC	3733.0 ± 171.0	2948.0 ± 640.3
	HMVEC	2855.0 ± 215.3	2032.0 ± 562.1
	BOEC	1336.0 ± 776.2	700.6 ± 536.1
Poly-IC HMW	AoEC	3407.0 ± 219.4	3933.0 ± 67.4
	HMVEC	3401.0 ± 357.8	3638.0 ± 239.3
	BOEC	3061.0 ± 330.1	3109.0 ± 770.2
IMQ	AoEC	262.0 ± 55.6	1743.0 ± 733.5
	HMVEC	127.8 ± 33.4	2151.0 ± 711.4
	BOEC	192.3 ± 39.2	1636.0 ± 592.4

Supplementary Table 1: IP-10 release from endothelial cells stimulated with PAMPs

IP-10 levels released in media from untreated (media only) or IL-1ß (10ng/ml) primed (3 hours) aortic (AoEC), microvascular (HMVEC) and blood outgrowth (BOEC) endothelial cells treated for 24 hours with control, LPS (1ug/ml), Poly-IC low or high molecular weight (LMW or HMW respectively; 10µg/ml) and Imiquimod (1µg/ml). Data are shown as the mean ± SEM from n=6 wells from n=3 separate donors for AoEC, HMVEC and BOEC. Data were analysed using a paired one-way.
