# Supplementary material for: Resistance of endothelial cells to SARS-CoV-2 infection in vitro
Source: J Virol. 2025 Dec 5;99(12):e01205-25. doi: 10.1128/jvi.01205-25 (PMC12724323; doi:10.1128/jvi.01205-25)
Supplement: Table S2 — Average raw Ct values from qRT-PCR. [file jvi.01205-25-s0007.pdf]

Gene	Cell type	Raw CT values		
**Donor/well**		**1**	**2**	**3**
ACE2	AoEC	38.3	37.5	36.2
	HMVEC	Undet.	Undet.	Undet.
	BOEC	Undet.	39.3	N/A
	NEC	31.3	31.2	30.7
TMPRSS2	AoEC	Undet.	Undet.	Undet.
	HMVEC	Undet.	Undet.	Undet.
	BOEC	Undet.	Undet.	Undet.
	NEC	29.2	27.6	27.5
BSG	AoEC	20.2	20.3	21.2
	HMVEC	23.2	24.3	21.0
	BOEC	23.4	20.4	N/A
	NEC	20.4	21.4	20.2
PPIA	AoEC	23.7	22.0	22.5
	HMVEC	23.9	23.6	22.8
	BOEC	24.1	22.0	N/A
	NEC	22.1	23.1	22.5
PPIB	AoEC	20.6	21.0	21.2
	HMVEC	24.1	24.0	22.2
	BOEC	24.2	21.2	N/A
	NEC	21.4	21.7	21.3
18S/GAPDH	AoEC	17.8	17.3	17.1
	HMVEC	19.1	20.0	17.9
	BOEC	21.0	16.7	N/A
	NEC	18.1	17.2	17.3

Supplementary Table 2: Average raw Ct values from qRT-PCR

Expression levels (Ct) for the genes *ACE2*, *TMPRSS2*, *BSG*, *PPIA* and *PPIB* were obtained from aortic (AoEC), microvascular (HMVEC) and blood outgrowth (BOEC) endothelial cells and nasal epithelial cells (NEC). Data for each donor were corrected using the average of the housekeepers (*18S* and *GAPDH*) and analysed using a comparative Ct method (2ΔΔCt). Data are shown as the mean +/- from n=3 wells using cells from 3 separate donors for AoEC and HMVEC and n=3 wells using cells from 2 separate donors for NEC and n=2 wells from 2 separate for BOECs.
